# 2746. Assessment of in vitro synergy between Piperacillin-Tazobactam-Meropenem and Imipenem-Meropenem against OXA-48 Producing *Klebsiella pneumoniae* Using Time-Kill Assays

**DOI:** 10.1093/ofid/ofad500.2357

**Published:** 2023-11-27

**Authors:** Mervenur Demir, Abdullah Tarik Aslan, Elif Seren Tanrıverdi, Barış Otlu, Murat Akova, David Paterson, Patrick N A Harris, Gülşen Hazırolan

**Affiliations:** Hacettepe University, Ankara, Ankara, Turkey; Hacettepe University, Ankara, Ankara, Turkey; Inonu University, Malatya, Malatya, Turkey; Malatya İnönü University, Malatya, Malatya, Turkey; Hacettepe University, Ankara, Ankara, Turkey; National University of Singapore; 5Pathology Queensland/University of Queensland, Brisbane, Queensland, Australia; Hacettepe University, Ankara, Ankara, Turkey

## Abstract

**Background:**

Although double carbapenem regimens have reliable synergy against carbapenemase-producing *Klebsiella pneumoniae* (CP-Kp), the capacity of piperacillin-tazobactam (TZP) to provide synergistic activity with meropenem (MEM) is uncertain.

**Methods:**

We used time-kill assays to evaluate synergy between TZP and MEM and imipenem (IMI)-MEM against non-clonal clinical CP-Kp isolates (tested by pulse-field gel electrophoresis). Five major carbapenemase genes (bla_KPC_, bla_NDM_, bla_IMP_, bla_VIM_, and bla_OXA-48 family_) were tested by an in-house PCR. The antibiotic concentrations used in the assays were determined in two ways. First, the concentrations were selected to reflect those achievable in vivo in critically ill patients using optimal dosing strategies, i.e., 16/4 mg/L for TZP, 8 mg/L for MEM, 4 mg/L for IMI. Second, for organisms in which the MEM MIC was < 8 mg/L and IMI MIC was < 4 mg/L, the time-kill assays were performed using concentrations of carbapenems relative to the isolate’s MIC. Synergy was defined as a ≥ 2-log_10_-CFU/ml reductions at 24 h of exposure to the antibiotic combination compared with the single most active antibiotic, with the number of surviving organisms in the presence of the combination at > 2 log_10_ CFU/ml below the starting inoculum.

**Results:**

17 OXA-48- and 3 NDM-1-producing Kp isolates were included. The combinations of TZP-MEM and IMI-MEM were synergistic against 41.2% (7/17) and 52.9% (9/17) of OXA-48 producers, respectively (Table 1, 2). The synergy between TZP and MEM (85.7%, 6/7) was relatively higher than that of IMI and MEM (71.4%, 5/7) for isolates with low MEM MIC (0.5-2 mg/L). However, the synergy was lower in TZP-MEM combination (10%, 1/10) compared to IMI-MEM combination (40%, 4/10) among isolates with MEM MIC ≥ 16 mg/L. The TZP and MEM combination was bactericidal, demonstrating a ≥ 4 log_10_ CFU/mL reduction at 24 h, among 35.3% (6/17) of all OXA-48 producers and 71.4% (5/7) of OXA-48 producers with low MEM MIC. In contrast, the bactericidal synergy between IMI and MEM was shown among 29.4% (5/17) of all OXA-48 producers and 28.6% (2/7) of OXA-48 producers with low MEM MIC.

There was no synergy against NDM producers for both combinations.

**Figure:**
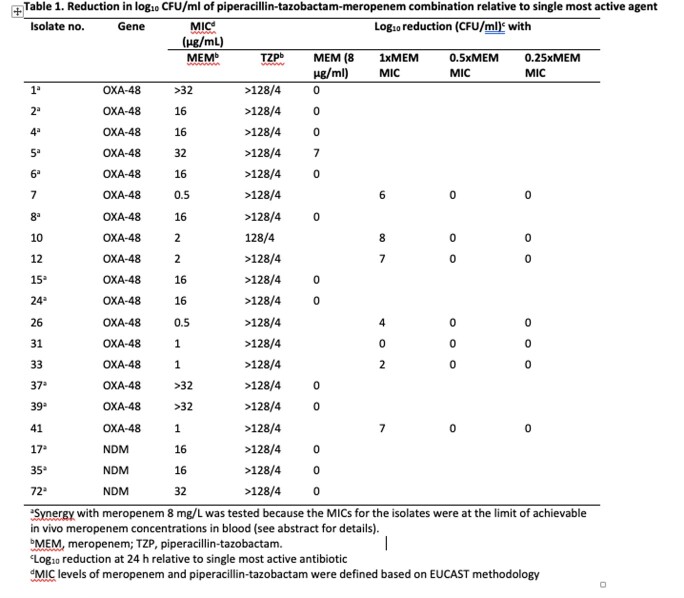

**Figure:**
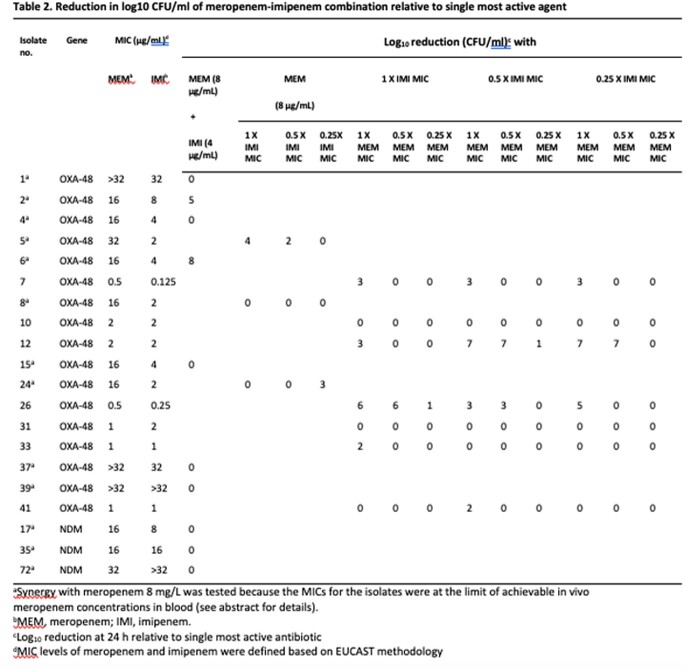

**Conclusion:**

TZP-MEM combination seems to be more reliable than double-carbapenem regimens for OXA-48-producing Kp with low MEM MIC.

**Disclosures:**

**David Paterson**, AMR Action Fund: Board Member|Entasis: Board Member|Merck: Board Member|Pfizer: Board Member|QPex: Board Member|Venatorx: Board Member **Patrick N A Harris, PhD, DTMH, MRCP, FRACP, FRCPA**, Merck: Advisor/Consultant|Opgen: Advisor/Consultant|Sandoz: Advisor/Consultant

